# Assessment of medical equipment maintenance management: proposed checklist using Iranian experience

**DOI:** 10.1186/s12938-021-00885-5

**Published:** 2021-05-21

**Authors:** Morteza Arab-Zozani, Ali Imani, Leila Doshmangir, Koustuv Dalal, Rona Bahreini

**Affiliations:** 1 Social Determinants of Health Research Center, Birjand University of Medical Sciences, Birjand, Iran; 2 Health Economics Department, Tabriz Health Services Management Research Center, School of Management and Medical Informatics, Tabriz University of Medical Sciences, Tabriz, Iran; 3 Tabriz Health Services Management Research Center, Iranian Center of Excellence in Health Management, School of Management and Medical Informatics, Tabriz University of Medical Sciences, Tabriz, Iran; 4 Department of Health Sciences (HLV), Public Health Science, Mid Sweden University, Sundsvall, Sweden; 5 Higher School of Public Health, Al Farabi Kazakh National University, Almaty, Kazakhstan; 6 Tabriz Health Services Management Research Center, Iranian Center of Excellence in Health Management, Student Research Committee, School of Management and Medical Informatics, Tabriz University of Medical Sciences, Tabriz, Iran

**Keywords:** Hospital, Medical equipment, Medical devices, Maintenance management, Assessment

## Abstract

**Background:**

Effective maintenance management of medical equipment is one of the major issues for quality of care, for providing cost-effective health services and for saving scarce resources. This study aimed to develop a comprehensive checklist for assessing the medical equipment maintenance management (MEMM) in the Iranian hospitals.

**Methods:**

This is a multi-methods study. First, data related to factors which affect the assessment of MEMM were collected through a systematic review in PubMed, ProQuest, Scopus, Embase, and web of science without any time limitation until October 2015, updated in June 2017. Then, we investigated these factors affecting using document review and interviews with experts in the Iranian hospitals. In the end, the results of the first and second stages were combined using content analysis and the final checklist was developed in a two-round Delphi.

**Results:**

Using a combination of factors extracted from the systematic and qualitative studies, the primary checklist was developed in the form of assessment checklists in seven dimensions. The final checklist includes 7 dimensions and 19 sub-categories: “resources = 3,” “quality control = 3,” “information bank = 4,” “education = 1,” “service = 3,” “inspection and preventive maintenance = 2” and “design and implementation = 3.”

**Conclusions:**

Developing an assessment checklist for MEMM provide a comprehensive framework for the proper implementation of accurate assessment of medical equipment maintenance. This checklist can be used to improve the profitability of health facilities and the reliability of medical equipment. In addition, it is implicated in the decision-making in support of selection, purchase, repair and maintenance of medical equipment, especially for capital equipment managers and medical engineers in hospitals and also for the assessment of this process.

## Background

Today’s modern hospital is highly dependent on various types of medical equipment to assist in the diagnosis, monitoring and treatment of patients. It is impractical to provide health services without them [1, 2]. Medical equipment deals with patient care including ranging from small and simple devices to complex and big devices. This ranking can be found in different types of hospitals and primary care settings [3]. According to the studies conducted in Iran, about one-third of the costs of setting up and equipping the hospital is allocated for purchasing medical equipment [4]. Therefore, it should be maintained in good working condition and higher safety level to prevent from injuries occurred in patients as well as in users [5].

The maintenance of medical equipment is important for reducing dispatch costs, reducing patient dissatisfaction, timely patient treatment, and reducing mortality and risks during patients care [4]. It is an integral part of the life cycle of the device. Usually, much more money is spent on maintaining equipment over than on its procurement [6]. Maintenance is defined as any action which helps hospitals to provide an adequate level of service and to protect or promote the performance of their equipment to operate regularly and efficiently. Therefore, maintenance management is a fundamental aspect of hospital management [7].

Good maintenance management to have well planned and implemented programs that hospitals can minimize breakdowns or failures of the medical device. This is particularly critical in developing countries for providing good healthcare services and saving scarce resources and alternatives. The equipment maintenance management of the hospital not only makes them easily accessible when needed but also increases their reliability and reduces their failure rate [4, 8]. Despite the importance of maintenance, there was no clear system of maintenance. The guidelines are not properly performed in many countries including Iran. In addition, there is a lack of information about the assessment and evaluation of medical equipment decisions [9].

A survey indicated that nearly 60% of the total cost of a hospital involves medical equipment [8]. Wang et al. have demonstrated that the most common cause of medical equipment downtime is poor maintenance, planning, and management [3]. One study indicates that nearly 1% of the total hospital budget is spent on maintenance costs [10]. The literatures have indicated that a 500-bed hospital spends typically around $5 million/year [6]. A study of world health organization (WHO) has shown that nearly half of medical devices in developing countries are operated incorrectly or are not maintained properly due to inappropriate management policy. On the other hand, the potential to manage and maintain medical equipment in these countries remains rather weak [11].

Medical equipment plays a significant role in the hospital system; hence, the purchase, maintenance and replacement of medical equipment are key factors in hospitals to implement medical care service. Thus, to assure the quality of healthcare delivery medical devices, use-safety assessment of the maintenance management in hospitals is imperative [12]. To achieve these objectives, hospitals must develop assessment checklists which identify the performance status of medical equipment maintenance. It is essential for managers and engineers, not only to enhance hospital capability but also to predict the risks related to sudden failure. Given the lack of single and comprehensive checklists for maintenance management, the purpose of the current research is to design and develop assessment checklists for medical equipment maintenance management (MEMM).

## Results

Of 309 potentially relevant articles searched, 29 articles were included in this systematic review. Finally, 89 factors were identified that affect the medical equipment maintenance management. These factors categorized based on MOHME framework [13]. Five of the factors were found related to resources, 12 factors related to service, 4 factors related to education, 15 of these factors regarding to quality control, 19 factors related to inspection, 12 factors related to information bank and 22 factors was dedicated to management [14].

The results of qualitative study were categorized into seven main themes (resources, quality control, documentation, education, service, inspection and preventive maintenance (IPM), designing and implementation) [15].

Based on the findings of the first and second steps, a medical equipment maintenance management assessment checklist was developed as follows (Table 1).

**Table 1:** Dimension of proposed medical equipment maintenance management assessment checklist

Sub-categories	Standards	Data collection method	Data collection source	Scores		
				0	1	2
Resources						
Physical resources	Providing appropriate physical space	Observation	Head of Medical Engineering Unit	Lack of space with adequate access	Enough space but inappropriate	Existence of a place with adequate and sufficient access
	Providing a special place for repairing devices in hospitals	Observation	Head of Medical Engineering Unit	There is no specific place		There is specific place
	Allocation of maintenance unit in medical engineering unit	Observation	Head of Medical Engineering Unit	The maintenance unit is not assigned to the medical engineering unit		The maintenance unit is assigned to the medical engineering unit
	Providing a safe and healthy work environment	Observation	Head of Medical Engineering Unit	The work environment is not safe and healthy		The work environment is safe and healthy
	Requirement of computer and necessary facilities in medical engineering unit	Observation	Head of Medical Engineering Unit	Lack of minimum necessary facilities (telephone line, fax and internet)	The necessary facilities (telephone line, fax and internet) are not enough	Equipped with telephone line, fax and internet, with the necessary administrative facilities
	Providing localized medical equipment maintenance software	Observation	Head of Medical Engineering Unit	The maintenance software available in the world is not used according to the native conditions of the country		The maintenance software available in the world is used according to the native conditions of the country
	The establishment of a maintenance computer system	Observation–documentation review	Head of Medical Engineering Unit	There are not enough conditions to set up a computer maintenance system (CMMS)		There are enough conditions to set up a computer maintenance system (CMMS)
	Provision of some technical checklists (test and repairs checklists)	Observation	Head of Medical Engineering Unit	Lack of checklists needed for safety testing and repairs	Incomplete availability of safety and repair test checklists	Existence of complete checklists required for safety test and repairs
	Existence of a specific system for recording failures	Observation–interview	Head of Medical Engineering Unit	There is no specific system for recording failures		There is specific system for recording failures
	Access to up-to-date software in the field of maintenance	Interview	Head of Medical Engineering Unit	It is not possible to access up-to-date software in the maintenance area		It is possible to access up-to-date software in the maintenance area
Human resources	Providing a stable and trained force for the medical engineering unit	Observation–interview	Head of Medical Engineering Unit—medical engineers and technicians	The available manpower has not been fixed and they have not received the necessary training	The available manpower has been fixed but has not received the necessary training OR The available manpower has received the necessary training but is not fixed	The available manpower has been fixed but has received the necessary training
	Allocation of medical engineering force based on need	Observation–interview	Head of Medical Engineering Unit	There are not enough skilled manpower (for every 100 hospital beds, at least one engineer and one technical technician)	There is skilled manpower, but not enough. (For every 100 hospital beds, at least one engineer and one technical technician)	There is enough skilled manpower. (For every 100 hospital beds, at least one engineer and one technical technician)
Financial resources	Allocation funds to medical equipment maintenance	Interview–documentation review	Head of Medical Engineering Unit	The required budget or credit is not estimated and provided each year in accordance with the Medical Equipment Maintenance Regulations		The required budget or credit is estimated and provided each year in accordance with the Medical Equipment Maintenance Regulations
	Assigning a revolving fund to the medical engineering unit	Interview–documentation review	Head of Medical Engineering Unit	The revolving fund is not allocated for the medical engineering unit		The revolving fund is allocated for the medical engineering unit
	Econometrics and cost–benefit calculation of equipment maintenance	Interview–documentation review	Head of Medical Engineering Unit	Documents are not available	Documents are incomplete	Documents are complete

Dimension	Standards	Data collection method	Data collection source	Scores		
				0	1	2
Quality control tests						
Safety test	Perform safety tests	Observation–interview	Head of Medical Engineering Unit	None of the safety tests (electrical, physical–mechanical, radiation, chemical, user, etc.) are performed according to the regulations for the maintenance of medical equipment	Some safety tests (electrical, physical–mechanical, radiation, chemical, user, etc.) are performed according to the regulations for the maintenance of medical equipment	All safety tests (electrical, physical–mechanical, radiation, chemical, user, etc.) are performed according to the regulations for maintaining medical equipment
Calibration	Ensuring quality controls performed by companies	Interview–documentation review	Head of Medical Engineering Unit	Quality controls performed by companies are not guaranteed (no specific obligations)		Quality controls performed by companies are guaranteed (have certain obligations)
	Planning and conducting quality control tests	Interview–documentation review	Head of Medical Engineering Unit	There are no specific program quality control tests		Quality control tests have a specific program
	Labeling of calibrated equipment	Observation–documentation review	Head of Medical Engineering Unit	Calibrated equipment is not regularly labeled	Calibrated equipment is labeled but not regularly	Calibrated equipment is regularly labeled
Performance test	Perform technical tests	Observation–documentation review	Head of Medical Engineering Unit	Technical tests are not performed by allowed agencies or service providers		Technical tests are not performed by allowed agencies or service providers
	Perform practical tests	Observation–documentation review	Head of Medical Engineering Unit	The relevant test is not performed by the trained user and is not according to the checklist provided by the manufacturer or legal representative and under the supervision of the Medical Engineering Unit		The relevant test is performed by the trained user and is according to the checklist provided by the manufacturer or legal representative and under the supervision of the Medical Engineering Unit
	Perform laboratory tests	Observation–documentation review	Head of Medical Engineering Unit	The relevant test is not performed by the trained user and is not according to the checklist provided by the manufacturer or legal representative and under the supervision of the Medical Engineering Unit		The relevant test is performed by the trained user and is according to the checklist provided by the manufacturer or legal representative and under the supervision of the Medical Engineering Unit
	Perform clinical tests	Observation–documentation review	Head of Medical Engineering Unit	The relevant test is not performed by the trained user and is not according to the checklist provided by the manufacturer or legal representative and under the supervision of the Medical Engineering Unit		The relevant test is performed by the trained user and is according to the checklist provided by the manufacturer or legal representative and under the supervision of the Medical Engineering Unit
Inspection and preventive maintenance (IPM)						
Management processes to extend the life of the device	Perform timely and regular preventive maintenance	Observation–documentation review	Head of Medical Engineering Unit	A preventive maintenance program is not developed and implemented	A preventive maintenance program is developed but it runs incompletely	A preventive maintenance program is developed and implemented
	Insufficient knowledge in the field of preventive maintenance	Interview	Head of Medical Engineering Unit—medical engineers and technicians	None of the employees of the Medical Engineering Unit have sufficient knowledge and awareness in the field of preventive maintenance	Some employees of the Medical Engineering Unit have sufficient knowledge and information in the field of preventive maintenance	All employees of the Medical Engineering Unit have sufficient knowledge and information in the field of preventive maintenance
	Preventive maintenance priority over quality control and repair	Interview	Head of Medical Engineering Unit	Preventive maintenance is not a priority in the medical engineering unit's action plan		Preventive maintenance is a priority in the medical engineering unit's action plan
Periodic, internal, case, practical inspection	Existence of some sub-rules	Interview	Head of Medical Engineering Unit	The existing rules and regulations regarding maintenance are not necessary and appropriate	The existing rules and regulations regarding maintenance are necessary but not appropriate	The existing rules and regulations regarding maintenance are necessary and appropriate
	Using accreditation measures to assess the maintenance of medical equipment	Interview	Head of Medical Engineering Unit	From accreditation measures are not used to assess maintenance activities and processes		From accreditation measures are not used to assess maintenance activities and processes
	Examination of medical equipment through human senses and specialized checklists	Interview	Head of the University Medical Equipment Office or Experts introduced by him and the head of the medical engineering unit	Lack of investigate and supervision of all operations (review and monitoring of quality control tests of medical equipment, review of facilities and ancillary facilities related to equipment, review of the environment around medical equipment, assessment of device usage by user and assessment of the performance of technical personnel) (By checklist)	Investigate and supervision of some operations (investigate and monitoring of quality control tests of medical equipment, investigate of facilities and ancillary facilities related to equipment, investigate of the environment around medical equipment, assessment of device usage by user and assessment of the performance of technical personnel) (By checklist)	Investigate and supervision of all operations (investigate and monitoring of quality control tests of medical equipment, investigate of facilities and ancillary facilities related to equipment, investigate of the environment around medical equipment, assessment of device usage by user and assessment of the performance of technical personnel) (By checklist)
	Determining the time period of periodic visits	Interview–documentation review	Head of Medical Engineering Unit	Not according to the action plan		According to the action plan
	Developing plan for periodic visits	Documentation review	Head of Medical Engineering Unit	An action plan has not been developed		An action plan has been developed
	Existence of an external observer	Interview	Head of Medical Engineering Unit and Hospital Manager	The external supervisor has not been appointed to inspect and oversee the affairs of the medical engineering unit		The external supervisor has been appointed to inspect and oversee the affairs of the medical engineering unit
	Developing standards in maintenance contracts	Documentation review	Head of Medical Engineering Unit	The service, maintenance and repair contract have not a specific framework. (According to regulations)		The service, maintenance and repair contract have a specific framework. (According to regulations)
	Preparing a list of essential equipment	Documentation review	Head of Medical Engineering Unit	The list of essential equipment has not been prepared and is not available to the departments	A list of essential equipment has been prepared but is not available to the departments	The list of essential equipment has been prepared and is available to the departments
	Ensuring the existence of safe electricity (every six months, the hospital's electricity is controlled by an electrician and the available documents)	Observation	Head of Medical Engineering Unit	The items mentioned in the regulations regarding electrical safety are not observed and performed	The items mentioned in the regulations related to electrical safety are incompletely observed and performed	The items mentioned in the regulations regarding electrical safety are completely observed and performed
	Periodic review of the correct operation of the device by the user	Interview–documentation review	Head of Medical Engineering Unit	The correct operation of the device is not checked periodically by the user		The correct operation of the device is checked periodically by the user
	Lack of uniformity of assessment checklists	Documentation review	Head of Medical Engineering Unit	The medical engineering unit does not have a specific checklist to assess the maintenance status of medical equipment		The medical engineering unit has a specific checklist to assess the maintenance status of medical equipment
	More visiting to defective devices	Interview–documentation review	Head of Medical Engineering Unit	Defective devices are not checked regularly by a medical engineer (existence of action plan)		Defective devices are checked regularly by a medical engineer (existence of action plan)
	Clarification of regulations and notification instructions	Interview–documentation review	Head of Medical Engineering Unit—medical engineers and technicians	None of the medical engineering staff are aware of the regulations and instructions issued by the Department of Medical Equipment/not all employees know the rules clearly	Some of the medical engineering staff are aware of the regulations and instructions issued by the Department of Medical Equipment/some of employees know the rules clearly	All of the medical engineering staff are aware of the regulations and instructions issued by the Department of Medical Equipment/all employees know the rules clearly
	Requirement to follow orders from high levels	Interview	Head of Medical Engineering Unit	Orders issued by the Department of Medical Equipment are not properly complied		Orders issued by the Department of Medical Equipment are properly complied
	Access to the booklet of the set of maintenance rules and regulations	Interview–documentation review	Head of Medical Engineering Unit	None of the staff members have access to the booklet	Some of the staff members have access to the booklet	All of the staff members have access to the booklet
	Completion and observance of accreditation measures	Interview	Head of Medical Engineering Unit	The unit does not meet any of the criteria on a regular basis	The unit meets some of the criteria on a regular basis	The unit meets any of the criteria on a regular basis
	Implementation of maintenance rules and regulations	Interview	Head of Medical Engineering Unit	The rules announced by the Office of Medical Equipment do not run regularly		The rules announced by the Office of Medical Equipment run regularly
	Existence of written and comprehensive instructions	Documentation review	Head of Medical Engineering Unit	There are no comprehensive guidelines for maintaining medical equipment	There are written guidelines for maintenance medical equipment, but they are not comprehensive	There are comprehensive guidelines for maintaining medical equipment
Information bank						
Providing medical devices ID (identification)	Electronic, comprehensive and intelligent electronic medical ID	Observation–documentation review	Head of Medical Engineering Unit	Medical ID are not regularly prepared and updated	Medical equipment ID has been prepared but not up to date	Medical ID are regularly prepared and updated
	Easy access to ID information	Interview–documentation review	Head of Medical Engineering Unit, Medical Engineers and Technical Technicians/Department Employees	Employees do not have access to ID information		Employees have access to ID information
Use of indigenous and global evidence	Communicate with standard scientific references	Interview–documentation review	Head of Medical Engineering Unit	The head of the medical engineering unit does not have access to scientific standard references (scientific articles, scientific sites, international standards, etc.)		The head of the medical engineering unit does not have access to scientific standard references (scientific articles, scientific sites, international standards, etc.)
	Utilizing the information of the manufacturer in the assessment	Interview	Head of Medical Engineering Unit	There is no access to the manufacturer’s information and it is not operated accordingly	There is access to the manufacturer’s information, but it is not operated accordingly	There is access to the manufacturer’s information and it is operated accordingly
	Conducting studies and research in the field of medical equipment maintenance	Documentation review	Head of Medical Engineering Unit	The medical engineering unit does not conduct research on the maintenance of medical equipment		The medical engineering unit conducts research on the maintenance of medical equipment
User guide	Preparing and installing quick user labels	Observation–documentation review	Head of Medical Engineering Unit	Quick user tags are not provided and installed on all devices		Quick user tags are provided and installed on all devices
	Preparation of manuals in both Persian and English for each device	Observation–documentation review	Head of Medical Engineering Unit	The manual of each device has not been prepared and has not been provided to the user of the device	The manual of each device has been prepared but has not been provided to the user of the device	The manual of each device has been prepared and provided to the user of the device
Documentation	Archive of executive processes (repair, quality control and PM documentation)	Observation–documentation review	Head of Medical Engineering Unit	Maintenance procedures are not fully documented and archived	Some of maintenance procedures are fully documented and archived	Maintenance procedures are fully documented and archived
	Use of HIS system in maintenance	Observation	Head of Medical Engineering Unit	The medical engineering unit is not equipped with the HIS system		The medical engineering unit is equipped with the HIS system
Educating						
User and technical education	Educational supervisor participation in user training	Interview–documentation review	Head of Medical Engineering Unit/Educational supervisor	Educational supervisors do not cooperate with medical engineers in educating users		Educational supervisors cooperate with medical engineers in educating users
	Targeted training courses	Interview	Head of Medical Engineering Unit, Medical Engineers and Technical Technicians/Department Employees	Training courses are not purposeful or useful		Training courses are purposeful or useful
	Acquaintance of the officials of the medical engineering unit with the management	Interview	Head of Medical Engineering Unit, Medical Engineers and Technical Technicians	None of the medical engineering officers are familiar with medical equipment maintenance management	Some of the medical engineering officers are familiar with medical equipment maintenance management	All of the medical engineering officers are familiar with medical equipment maintenance management
	The effectiveness of training courses	Interview	Head of Medical Engineering Unit, Medical Engineers and Technical Technicians/Department Employees	The trainings are not effective		The trainings are effective
	Providing the possibility for all medical engineers to participate in training workshops	Interview	Head of Medical Engineering Unit, Medical Engineers and Technical Technicians	It is not possible for all engineers and technicians to participate in training workshops		It is possible for all engineers and technicians to participate in training workshops
	Nurse’ level of education in the field of general equipment	Interview	Users	Medical equipment users have not been trained in general equipment		Medical equipment users have not been trained in general equipment
	User retraining about essential equipment	Interview	Users	Users are not retrained about essential equipment		Users are retrained about essential equipment
	Invitation of expert professors to teach	Interview	Head of Medical Engineering Unit	Expert professors are not invited to teach		Expert professors are invited to teach
	Visiting the production lines of medical equipment and supplies companies	Observation	Head of Medical Engineering Unit, Medical Engineers and Technical Technicians	Medical engineers and technicians do not visit manufacturing companies		Medical engineers and technicians visit manufacturing companies
	Participate in in-service training for official and contract forces	Interview–documentation review	Head of Medical Engineering Unit, Medical Engineers and Technical Technicians	Medical engineers and technicians do not participate in in-service training		Medical engineers and technicians participate in in-service training
	Conducting classes and training courses for managers, engineers and users	Interview–documentation review	Head of Medical Engineering Unit, Medical Engineers and Technical Technicians/Users	Classes and training courses are not scheduled		Classes and training courses are held as scheduled
	Developing a training program based on the training needs of users and engineers	Interview–documentation review	Head of Medical Engineering Unit	The training program is not developed based on educational needs		The training program is developed based on educational needs
Service						
Repair (corrective maintenance)	Repair of medical equipment according to the regulations and supervision of device repair	Interview	Head of Medical Engineering Unit	The repair of medical equipment is not in accordance with the regulations and the medical engineer does not observe their repair by companies	Medical equipment is repaired according to the regulations, but the medical engineer does not observe their repair by companies	The repair of medical equipment is in accordance with the regulations and the medical engineer does observe their repair by companies
	Necessity of warranty and after-sales service in repair	Documentation review	Head of Medical Engineering Unit	Medical equipment has no warranty or after-sales service	Some medical devices have a warranty and after-sales service	Medical equipment has warranty or after-sales service
	Repair of vital and capital equipment by allowed agencies	Interview–documentation review	Head of Medical Engineering Unit	Capital equipment is not repaired by allowed agencies	Some of the capital equipment is repaired by allowed agencies	Capital equipment is repaired by allowed agencies
	Make it possible to repair the device inside the hospital	Interview	Head of Medical Engineering Unit	It is not possible to repair some medical equipment inside the hospital		It is possible to repair some medical equipment inside the hospital
Decommissioning	Observing the decommissioning process in maintenance management	Observation–documentation review–interview	Head of Medical Engineering Unit	The decommissioning process is not followed according to the instructions and notification rules		The decommissioning process is followed according to the instructions and notification rules
Outsourcing	Communication with reputable companies	Interview–documentation review	Head of Medical Engineering Unit	Medical engineering unit is not associated with reputable companies		Medical engineering unit is associated with reputable companies
	investigate the performance of private companies	Interview	Head of Medical Engineering Unit	The performance of private companies is not monitored by the Department of Medical Equipment		The performance of private companies is monitored by the Department of Medical Equipment
	The role of third parties in quality control	Interview	Head of Medical Engineering Unit	Quality control is not done by private companies		Quality control is done by private companies
	Buy maintenance software from private companies	Observation–documentation review	Head of Medical Engineering Unit	Maintenance software is not purchased from private companies		Maintenance software is purchased from private companies
	Cooperation and coordination of ministries and universities with private companies	Interview	Ministry and university officials	The Ministry and the University do not cooperate with private companies		The Ministry and the University cooperate with private companies
	Outsourcing of service, maintenance and repair	Documentation review	Head of Medical Engineering Unit	Outsourcing does not have a general framework according to the regulations		Outsourcing does not have a general framework according to the regulations
Designing and implementation						
Process management	Formation of joint committees by the Department of Medical Equipment	Documentation review	Head of Medical Engineering Unit	The committee’s minutes are not available	The minutes of the committee are available, but not complete	The minutes of the committee are complete
	Define the level of user access to medical equipment	Interview	Head of Medical Engineering Unit, Medical Engineers and Technical Technicians/Users	The user access level policy is not defined		The user access level is defined according to the relevant policy
	Inter-sectorial communication in hospitals	Interview–observation	Head of Medical Engineering Unit, Medical Engineers and Technical Technicians/Users	The Medical Engineering Unit does not have a specific protocol for communicating with other departments and units		The Medical Engineering Unit has a specific protocol for communicating with other departments and units
	Planning for maintenance in the office of medical equipment	Interview–documentation review	Head of Medical Equipment Department	The Medical Engineering Unit has no plans for any medical equipment maintenance activities	The Medical Engineering Unit has plans for some medical equipment maintenance activities	The Medical Engineering Unit has plans for all medical equipment maintenance activities
	Develop and define internal policies	Documentation review	Head of Medical Engineering Unit	No policy has been developed for the maintenance of medical equipment		A policy has been developed to maintenance medical equipment
	Develop an action plan for the medical engineering unit	Documentation review	Head of Medical Engineering Unit	An action plan for maintaining medical equipment has not been developed		An action plan for maintaining medical equipment has been developed
	Management infrastructure in the medical equipment department	Interview	Head of Medical Equipment Department/Head of Medical Engineering Unit	The Department of Medical Equipment does not have a strong infrastructure		The Department of Medical Equipment have a strong infrastructure
	Existence of a specific assessment structure for maintaining medical equipment	Interview–observation	Head of Medical Equipment Department/Head of Medical Engineering Unit	Medical equipment maintenance does not have a clear structure for assessing this process		Medical equipment maintenance does not have a clear structure for assessing this process
	Prolonged administrative processes	Interview	Head of Medical Equipment Department/Head of Medical Engineering Unit	The administrative process is long and cumbersome		The administrative process is not long and cumbersome
	Creating unity of procedure in the medical engineering unit	Interview–observation	Head of Medical Engineering Unit	The Medical Engineering Unit does not have the unity of procedure in the implementation of affairs		The Medical Engineering Unit has the unity of procedure in the implementation of affairs
Knowledge and attitude	Medical professional dominance in hospitals	Interview	Head of Medical Equipment Department/Head of Medical Engineering Unit	There is Medical professional dominance in hospitals to buy and choose medical equipment		There is not Medical professional dominance in hospitals to buy and choose medical equipment
	Involvement of non-experts in the medical equipment department	Interview	Head of Medical Equipment Department/Head of Medical Engineering Unit	Non-experts are involved in decision-making and policy-making in the field of medical equipment		Experts are involved in decision-making and policy making in the field of medical equipment
	The correct understanding of management and users hospital about maintenance management	Interview	Hospital management and users	Hospital management and users are not familiar with maintenance management		Hospital management and users are familiar with maintenance management
	Change the view of the officials to the medical engineering unit	Interview	Head of Medical Equipment Department/Head of Medical Engineering Unit	The medical engineering unit in the hospital does not have a specific and established position		The medical engineering unit in the hospital has a specific and established position
Purchasing management	Buy the device from the website of reputable companies	Documentation review	Head of Medical Equipment Department/Head of Medical Engineering Unit	Medical equipment is not purchased from reputable companies		Medical equipment is purchased from reputable companies
	To buy equipment based on need	Documentation review	Head of Medical Equipment Department/Head of Medical Engineering Unit	Medical equipment is not purchased based on the needs of the area and the target		Medical equipment is purchased based on the needs of the area and the target

The checklists have seven dimensions, each of which includes sub-categories, such as the provision of variety of financial, human and physical resources, which means physical resources to provide a safe and secure environment equipped with the necessary facilities. Human resources refer to the provision and allocation of experienced and skilled manpower based on need. Financial resources also include the allocation of sufficient and necessary funds and budgets, which should be based on the goal and be allocated to priority goals according to the operational plan of the medical engineer unit.

The dimension of quality control tests in the three sub-categories of safety test, performance test and calibration refer to all technical tests that require special equipment and are of special importance for the health of the patient and staff. The Inspection and Preventive Maintenance Item refers to the importance and priority of PM than repairing and assessing the user and performance of personnel. In this section, there are topics such as periodic inspections, development of maintenance standards, the existence of external supervisors, and the existence of written and comprehensive guidelines.

In the field of information bank, all activities related to the process of documentation and identification of medical equipment is listed. In the training section, both technical and user training for medical engineers and users are mentioned. The sub-categories of the service sector include after-sales service, repair and maintenance contracts, outsourcing of the decommissioning process, and so on. The last dimension is design and implementation, which refers to issues such as defining the level of user access, organizing joint committees, establishing inter-sectorial communication, policy development, purchasing medical equipment based on needs, and so on.

The scoring method is in three categories of zero, one and two. If the item meets the criteria, score 2, in case of partial compliance, one score and in case of mismatch, no points are awarded. For some items, one score is not included. That is, only two matching or mismatch modes are applicable and no intermediate states. Part of the checklist is about data collection that depending on the nature of the item, the collection method involves interview, observation and documentation review. Thus, the interview will be used if we are looking for the views and attitudes of stakeholders. Dimensions that require documentation will be assessed through documentation review. The observation method is also for assessing physical items.

## Discussion

In the field of medical equipment maintenance management, there is no single and standard checklist that includes all hospitals in the country. The only available checklists include accreditation measures that generally assess the tasks and activities of the medical engineering unit. Since the issue of maintenance management includes a wide variety of topics, and in the low- and middle-income countries, there is major weakness in this regard, so we decided to design a checklist for uniformity and accurate and comprehensive assessment using Iranian context. The Departments of Medical Equipment of some provinces in Iran have designed a checklist natively for its affiliated hospitals, the dimension and method of which are different. For example, the maintenance management evaluation checklist of Tabriz Medical Equipment Office includes 15 indicators (technical force, medical engineering unit, medical equipment ID, quality control tests, PM, training, medical equipment and spare parts storage, service and maintenance contract, the existence of purchase process, the existence of decommissioning process, the existence of recall system and reporting of adverse events, ensuring sound electricity, implementing a continuous maintenance improvement process, familiarizing with the general administration’s rules and website, management and allocating a separate budget for maintenance).

According to the medical equipment maintenance management criteria of the MOHME, some of these dimensions can be merged into one dimension and some can be separated. In addition, each of the indicators and sub-indicators can be expanded. That is, not all maintenance management issues are addressed. The scoring and classification of dimensions in this checklist does not have a specific standard and does not include all dimensions of maintenance according to the maintenance criteria of the MOHME and is generally designed.

Herrera-Galán [16] evaluated the performance of the maintenance function through management audits and their implementation in five hospitals. The aspects evaluated include equipment availability, response to a service request, monitoring and control of biomedical equipment, staff training, quality of work executed by the maintenance technicians, the workload of maintenance technicians, control of the work executed by the maintenance technicians, the effectiveness of annual maintenance planning and department performance. The results of this research show that the audit technique is a valuable checklist in the performance assessment of a hospitals. The application of the proposed method evidenced that the most critical component in the results of a management audit is the human resource [16].

An effective medical equipment maintenance program consists of three main elements. (1) Identifying the medical devices that need to maintenance program by the Ministry of Health. (2) Financial management, personal management, performance monitoring, operational management and performance improvement. (3) Proper implementation of the maintenance program. These three elements are also considered in the designed checklists in different dimensions such as resources, designing and implementation [17].

The maintenance and its management constitute a checklist that ensures the equipment performance. There exist four criteria in which the hospitals coincide that they should improve, even though each of them in different measure and sub-criterion. These criteria are an organization of maintenance; human resources; planning, programming and control of the maintenance and corrective maintenance. These criteria are among the sub-categories of maintenance management assessment checklists [18].

Amerion et al. [19] identified effective factors on the MEMM in a military hospital. Among effective factors on the MEMM, 26 components were extracted. User training components, human resources, commitment and the experience of users, the foreign exchange market, periodic visits, and trade name were the most important components which had more than 75% of the relative abundance. According to the results, factors with high importance on the management of medical equipment maintenance should be supported by the center’s directors. Attention to the use of these components can reduce maintenance costs, and therefore, increase the life of medical equipment. User training and human resources are the two main dimensions of this checklist [19].

According to the current results, documentation and service are two dimensions of MEMM. The problems of some hospitals in MEMM were introduced from the aspects of maintenance time, maintenance record, maintenance service and self-maintenance. Some measures were proposed including simplifying maintenance process through PDCA, information maintenance record, cooperating with the third-party maintainer and establishing self-maintenance team, so that precision and information medical equipment management can be realized to maximize the benefit of medical equipment management [20].

The medical equipment requires maintenance (both scheduled and unscheduled) during its useful life. The medical equipment maintenance process should be planned, implemented, monitored, and improved continually. This process requires careful supervision by healthcare administrators, many of whom may not have the technical background to understand all of the relevant factors. Maintenance management is the most important function in overall medical equipment management. In this regard, implementation of appropriate maintenance strategies requires the following types of resources: human resources, material resources, financial resources and documentation. Our findings also point to the importance of these resources [21].

We need a comprehensive assessment checklist that covers all aspects of medical equipment maintenance management in hospitals. In this regard, the identification of influential factors is essential. Eighty-nine factors were identified that affect MEMM. Five of the factors were found related to resources item, 12 factors related to service, 4 factors related to education, 15 of these factors regarding quality control, 19 factors related to inspection, 12 factors related to information bank and 22 factors were dedicated to management. These factors are implicated in decision-making in support of selection, purchase, repair and maintenance of medical equipment, especially for capital equipment managers and medical engineers in hospitals and also for the assessment of this process. Identification and classification of influential factors can be of help for raising critical alerts about the types of equipment more prone to maintenance problems [14].

## Strengths and limitations

In our knowledge, this study is the first comprehensive study of-its-kind addressing all of the factors affecting an effective and efficient MEMM. It offers comprehensive checklists for assessing the status of MEMM. In addition, the present compiled checklist is the result of multi-method research in such a way that its components are determined through systematic review and obtaining the views of experts and specialists in this field. This indicates the validity and comprehensiveness of the checklists developed.

Lack of enough information concerning the concept of maintenance management, lack of specific guidelines and instructions were the most notable limitations.

## Conclusion

Effective maintenance management of medical devices increases the efficiency and productivity of health technology resources, which is especially important when resources are limited. This allows patients to access medical equipment that can provide an accurate diagnosis, effective treatment, or appropriate rehabilitation. Several factors affect the management of medical equipment maintenance, and it is important to follow each of them to improve the performance of devices and provide medical services to patients. Therefore, before designing an assessment checklist, we need to consider a specific framework for the maintenance management process to include all maintenance activities. The medical equipment maintenance management assessment checklists allow for the timely identification of deficiencies and gaps in the medical equipment maintenance management process so that the necessary steps can be taken. It also can help managers and engineers to assess maintenance status and provide solutions and interventions for the decision makers and policymakers to improve its.

## Methods

The present study was a multi-methods study in four stages. In the first stage, a systematic review on data related to factors which affect the assessment of MEMM was conducted. Then, a qualitative study was designed to investigate these factors from expert view and related documents. In the end, the results of the first and second stages were combined using content analysis and the final checklist was developed.

### Stage 1

A systematic search of the following databases was conducted during October 2015 in the PubMed, ProQuest, Scopus, Embase, and Web of Science. The search was updated in June 2017. Our search strategy was as follow: ((“medical device*”[Title/Abstract]) OR “medical equipment”[Title/Abstract]) AND “maintenance management”[Title/Abstract])) [14]. At first, we extracted the items, then categorized the extracted items based on the Ministry of Health and Medical Education of Iran (MOHME) framework in each category [13].

### Stage 2

In the qualitative step, semi-structured interviews and documents review were used for data collection. The collected data consisted of the perspectives of medical and biomedical engineers concerning factors influencing MEMM. Related documents were regulations of MEMM, MEMM guidelines and other related regulations or reports developed by MOHME. The content analysis approach (inductive and deductive) was used to analyze the data. The extracted codes were sorted into both themes and subthemes based on comparisons between similarities and differences that were categorized into 7 main themes and 22 subthemes [15].

### Stages 3

In this stage, the researchers combined the extracted data from systematic review and qualitative study using a content analysis and devised the first draft of the checklist. The primary checklist was developed in the form of assessment checklists in seven dimensions (providing resources, quality control tests, preventive inspection and maintenance, database, training, service, design and implementation).

### Stages 4

We sent the primary draft of checklist through a Delphi to 20 experts who had sufficient scientific and information background in the field of MEMM for assessing the validity and reliability. Of them seven experts participated in this stages and send their comments. In the first round, experts commented on the content, dimensions, writing and appearance of the primary checklist. The research team modified the primary version based on expert’s comments and sends it again to experts. Eventually, the proposed checklist was finalized after approving the experts.

## Data Availability

The datasets of this study are available from the corresponding authors upon reasonable request.
